# Predictions of coronavirus COVID-19 distinct cases in Pakistan through an artificial neural network

**DOI:** 10.1017/S0950268820002174

**Published:** 2020-09-21

**Authors:** Iftikhar Ahmad, Syed Muhammad Asad

**Affiliations:** Department of Mathematics, University of Gujrat, Gujrat, Pakistan

**Keywords:** Artificial neural network, COVID-19, dataset, Pakistan, pandemic, predictions

## Abstract

This study presents the main motivation to investigate the COVID-19 pandemic, a major threat to the whole world from the day when it first emerged in China city of Wuhan. Predictions on the number of cases of COVID-19 are crucial in order to prevent and control the outbreak. In this research study, an artificial neural network with rectifying linear unit-based technique is implemented to predict the number of deaths, recovered and confirmed cases of COVID-19 in Pakistan by using previous data of 137 days of COVID-19 cases from the day 25 February 2020 when the first two cases were confirmed, until 10 July 2020. The collected data were divided into training and test data which were used to test the efficiency of the proposed technique. Furthermore, future predictions have been made by the proposed technique for the next 7 days while training the model on whole available data.

## Introduction

A novel coronavirus pandemic known as COVID-19 is an infectious disease which has become a major threat throughout the world since the date it first emerged in November 2019 in China city of Wuhan [1, 2]. Later, the disease spread throughout the world and as of 11 July 2020 more than 12.6 million cases has been confirmed in 213 countries, territories and two international conveyances resulting in 562 039 deaths and 7.3 million recovered people [3]. Initially, COVID-19 causes various degrees of illness affecting the human respiratory system [4]. Since there is not enough knowledge about the disease and no vaccine has been discovered until now, the pandemic is worst affecting the whole world economy, people daily life forbidden inside homes, institutions, industries and markets are disturbed because governments took necessary measurements to prevent its spread and decrease the number of affected cases. World Health Organization (WHO) has recommended several precaution measures including the physical distance between people, wearing masks and sanitising the hands to prevent and slow down the spread of the pandemic [5].

### COVID-19 in Pakistan

Pakistan has also been affected by this pandemic from the last few months in 2020. On 26 February 2020, Pakistan confirmed its first two cases, one in Karachi and the other one in federal areas of Islamabad; both of them had travelled from Iran [6]. On 18 March 2020, the first death in Pakistan was reported in Mardan, Khyber Pakhtunkhwa where the total number of cases was 302 [7]. To prevent the outbreak in the country, the government of Pakistan took several steps. First, a system was established to screen every passenger who had a travel history from most affected countries such as China, Iran, South Korea, etc., [8]. Based on the collected data on 10 July 2020, total number of confirmed cases were 246 351 [9]. In order to observe the behaviour of the graph of increasing cases in the country, it is very important to know about the number of increasing cases in the coming days so that essential measurements can be taken. For this purpose, an artificial neural network (ANN) technique has been used in this research.

### Artificial neural network

ANN is a biologically inspired computing system in which a connection system is made which learns by different techniques inspired by human learning and predicts future data. This topic was first developed by Warren Mc-Cullock and Walter Pits in 1943 by creating a model for ANN [10]. ANNs are sometimes more magnificent to make predictions where humans are not, e.g. decision support in cancer, streamflow forecasting, weather forecasting, etc. [11–13]. An ANN is constructed by the connection of units called neurons which can transmit signals to other neurons. An ANN can be made by many neurons working collectively to predict the future. A neuron or group of neurons make a layer and an ANN can have many such layers. A simple ANN can have one input layer which receives the input data and an output layer which gives results or predictions and one may have one or more hidden layers between input and output layers which can be used to transform input data to something which can be used by output layer to predict more accurate results. Data are passed in the form of real numbers to the input layer. Each connection between two neurons has a weight value which is optimised through training.

Once a neuron receives inputs from all the connected neurons in previous layers, accepting neuron adds its bias and applies an activation function and generates an output value. The activation function of a neuron is an actual power of an ANN. Activation functions are selected based on the problem or the one which is found to be more suitable during experiments. Different types of ANN models are trained through different techniques. Some further characteristics of an ANN are as below:

- ANNs have ability to learn from given data and adjust their weights accordingly in order to predict data which have not observed before.
- The learned parameters of an ANN are stored in its own network and therefore no databases are needed and hence these are not affected by loss of data.
- Similar to human, ANNs learn from examples. E.g. an ANN can be used to classify images like cats and dogs. Initially, the ANN is trained on some examples of cats and dogs images and then it could be able to classify those cats and dogs images which were not available during training [14].
- Unlike human, ANNs can be used to predict information about coming days e.g. ANNs are used widely in weather forecasting [13].

A brief description of supervised and unsupervised learning is discussed in [15]. In this research study, a supervised ANN has been used. Some of the characteristics of this study are summarised as follows:

- A novel design of an ANN is implemented to predict the number of deaths, recovered and confirmed cases of COVID-19 in Pakistan by using previous data of 137 days of COVID-19 cases in Pakistan.
- The data collected were divided into two main groups, one is training and the other is testing data, which are used to test the efficiency of the proposed techniques through a Python (Computer Programming Language CPL) library Tensor flow.
- The competency of computational numerical solver based on a rectifying linear unit-based technique is used for the dynamical study of the COVID-19 cases in Pakistan in terms of sufficient graphical and numerical representations.

A brief description of supervised and unsupervised learning is discussed in [15]. For recent studies on algorithms of numerical computing, the reader is referred to [16–19]. In this research study, a supervised ANN has been used. The rest of the paper is organised as follows: method used in this study is discussed in Section ‘Methodology’; modelling is presented in Section ‘Modelling of ANN’, ‘Simulation and Predictions’ Section presents the simulation and predictions of the proposed model and conclusion and future recommendations are discussed in the last section.

## Methodology

**Fig. 1.**
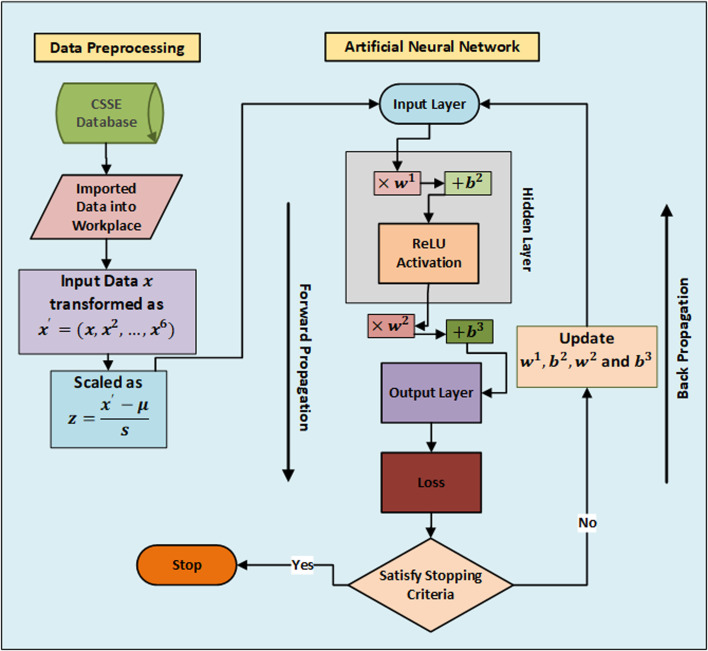
Flowchart of the proposed methodology.

In this research study, an ANN is used to make predictions of increasing rate of coronavirus confirmed cases, deaths and number of people who have recovered from this disease. A supervised ANN is used which is trained based on the collected data of past few days. A flowchart to represent the proposed methodology is given in Figure 1. This section is divided into three subsections: first, discussed about the data gathering sources and characteristic of data. Second, feature engineering and data preprocessing is performed to make data well suitable for neural network training and predictions. Third, a brief description on mathematical background of the ANN is discussed.

### Data gathering

**Fig. 2.**
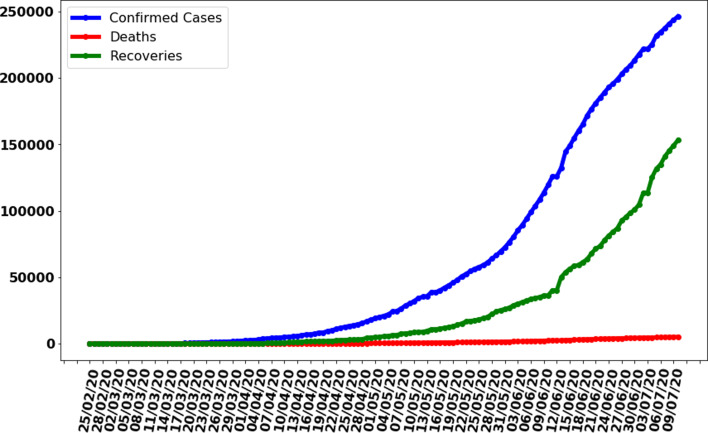
Cases recorded in Pakistan from 25 February 2020 to 10 July 2020.

The data gathered in this research study have been collected from the repository of Centre for System Science and Engineering (CSSE) at Johns Hopkins University and also supported by ESRI Living Atlas Team and a semi-automated living data stream strategy has been used to update data automatically during whole day with time interval of 15 min [20]. The data have provided for the whole world but in this research study, we filtered data for Pakistan only. Also, the data source confirmed that the data are collected from the Ministry of National Health Services Regulation and Coordination, Government of Pakistan official website [9]. We also compared the collected data with the graphs available at the Government of Pakistan official website for COVID-19 statistics and a perfect match was obtained. The data have divided into three different categories with time interval of 24 h (a day). First, it contains the cumulative total number of confirmed cases of coronavirus-infected people increasing daily; second, the total counted deaths due to the virus and third is those who have recovered from the virus and discharged from hospitals. A graph representing the characteristic of data is shown in Figure 2 for all three types of data, i.e. confirmed cases, deaths and recoveries from 25 February 2020 to 10 July 2020. It can be observed that the number of confirmed cases and total number of recoveries are increasing exponentially with 246 351 and 153 134 reported on 10 July 2020 respectively. Also, the total number of deaths is increasing and to 10 July 2020 total number of deaths counted were 5123. It was also observed that the average of confirmed cases, deaths and recoveries was 1798, 37 and 1117 respectively based on the collected data of 137 days.

### Data preprocessing

Once we have collected data, next step is preprocessing of data. Data preprocessing is an important part of machine learning to obtain better results. For this whole section, a Python (Computer Programming Language CPL) library Scikit-Learn is used [21]. As we can observe that the input of data is just dates in which cases were recorded while output is the amount of cases. Any machine-learning algorithms use numeric values for processing. Therefore, data featuring was performed and date values were transformed to integer values for the number of days from 1 to 137. After data featuring, a simple problem for the prediction of cases can be written as1
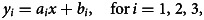
where *a_i_* and *b_i_* are coefficients or weights to be adjusted, *x* is the input value (number of days) and *y_i_* represent confirmed cases, deaths and recovered people. Although equation (1) is an equation of straight line, from Figure 2, it can be observed that the graph is not a linear. Therefore, to overcome this issue, the data preprocessing for the polynomial regression model technique is used and the equation was transformed into following polynomial equation:2
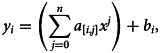
where *n* is the degree of polynomial which can adjust by experiments. In this research study, it is observed that *n* = 6 predicting better results with good accuracy along actual case curves. Based on this concept input data with one input value is transformed into six input values as follows while skipping constant value:3
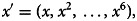
where *x*^′^ is the resulting vector for each input value *x*. For training and validation of the ANN, both the input and output data are split into train and test sets with the ratio of test size of 0.05 and without shuffling the data. Later, for predictions in the coming days, the model will be trained on whole data. After the transformation of input data of 137 samples into polynomial problem data, it can be observed that the input data values range between the interval [1, 3 138 428 376 721] which is a very large input value. The ANN is good to make predictions with standardised data which was centred around 0 and have variance in the same order. A data scaling was performed on input data as follows:4
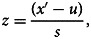
where *u* is the mean and *s* is the standard deviation of all samples of respective input values. After completing data preprocessing, next step is to design an ANN model which could predict better results.

## Modelling of ANN

**Fig. 3.**
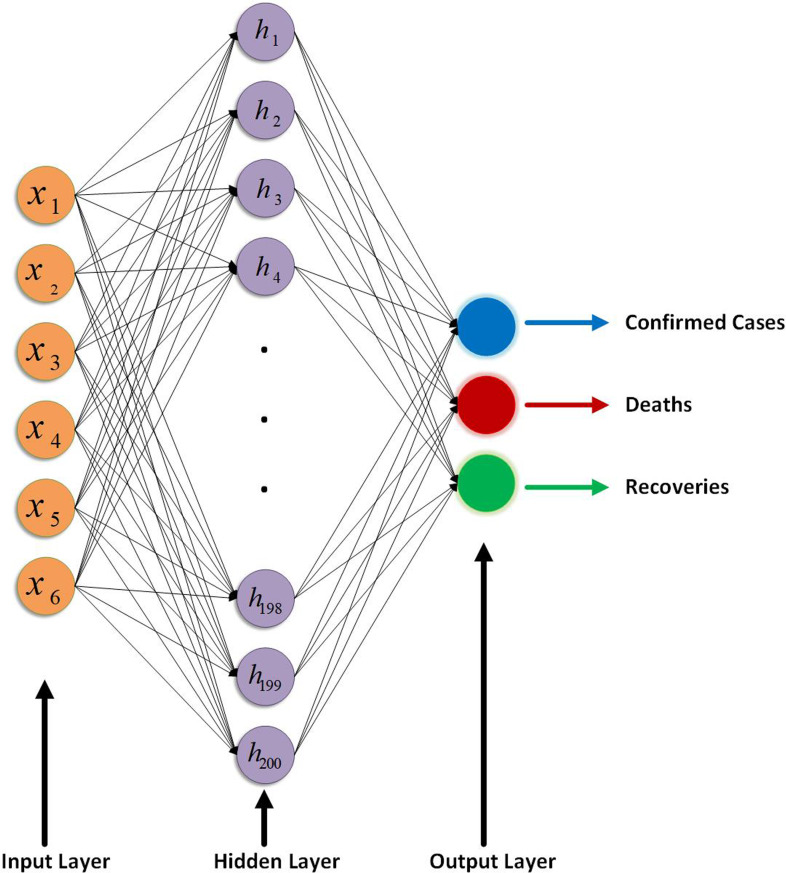
Graphical representation of the proposed ANN.

**Fig. 4.**
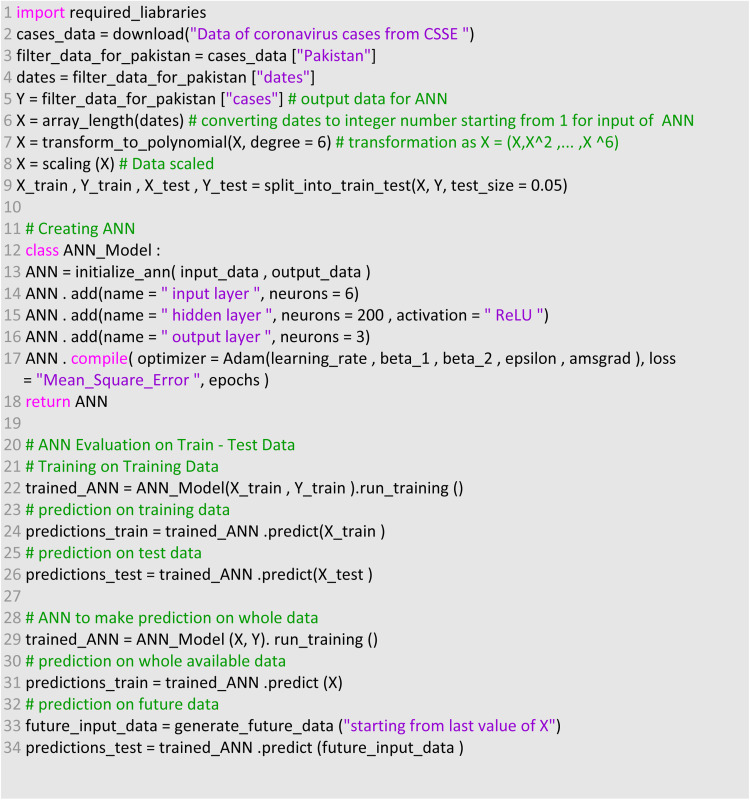
Pseudo-code of methodology.

In this section, a brief discussion on the modelling of proposed ANN is given. For whole work of this section, TensorFlow 2.0 is used which is an interface for expressing, implementing and deployment of machine-learning models on heterogeneous systems and it is available with Python CPL [22]. The proposed model has designed with three layers, input, output and hidden layers. The input layer has six neurons as data were transformed into (6 × No. of Samples) size as discussed in Section ‘Data preprocessing’ and in equation (3). Output layer has three neurons: one is predicting total number of confirmed cases, second is predicting deaths and third predicting total number of recovered people. Hidden layer has 200 neurons and in the hidden layer nodes a rectifying linear activation function is used. A graphical representation of the proposed ANN is shown in Figure 3 with six neurons in the input layer, 200 neurons in hidden layer while three neurons in the output layer where hidden and output layer are deeply connected with the previous layer. A mathematical formulation for proposed ANN is given as5
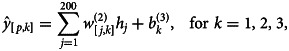
where *p* represents the number of samples from dataset fed into the network, *j* is the number of neurons in the hidden layer and *k* is the number of neurons in the output layer. 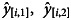 and 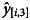 are the predicted values of the confirmed cases, deaths and recoveries against each *i*th input. Sum of 200 represents 200 hidden layer neurons, *w* are weights and *b* are biases. Superscripts of *w* and *b* represent the number of hidden layers e.g. 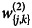 are weights of the second layer between each *j*th hidden layer neuron and *k*th output layer neuron. *h_j_* is the output of each *j*th hidden layer and defined as6
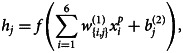
where 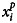 is input value of *i*th input neuron of sample *p*. *f* is an activation function of hidden neurons and as mentioned earlier a rectified linear unit (ReLU) activation is used in this ANN model, which is a piecewise function and return positive value i.e. 0 if input value is negative and can be defined as7
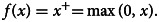
The ReLU activation function was first introduced by Hahnloser *et al*. in 2000 and a brief discussion and mathematical justification of this activation function can be found in their studies [23, 24]. Also, it was observed that, in the proposed ANN model 2003 are trainable parameters where 1400 are between the input and hidden layers and 603 between the hidden and output layers. After completing the design of the ANN model, next step was to train the model. The pseudo-code of the proposed scheme is shown in Figure 4. For the training of the ANN model, an Adam optimiser was used which is a stochastic gradient descent method for first-order gradient-based optimisations. This optimisation algorithm is well suitable for those problems which are large in terms of data or parameters or both of them. This optimisation algorithm is an extension of stochastic gradient descent algorithm, and it takes benefits from the Adaptive Gradient Algorithm (AdaGrad) and Root Mean Square Propagation (RMSprop).

The Adam algorithm makes use of exponentially weighted average and the decay rate is controlled by beta 1. It also calculates the average of the second moment of gradient and parameter for this decay rate is beta 2. Many authors have proposed their research studies on improvement and performance of Adam algorithm and is widely used by scientists in the field of ANN [25–28]. This optimisation algorithm was introduced by Diederik P. Kingma and Jimmy Lei Ba in 2014 and complete details can be found in [29]. In the proposed ANN, objective function was mean square error (MSE) between the predicted and actual values which were optimised through Adam. The mathematical notation of the performance metrics through MSE is given as below:8
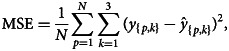
where *N* is the total number of samples in the training dataset. A parameter setting for optimisation of ANN is given in Table 1 which influence the performance of ANN. A well setting of parameters was obtained through experiments and literature. The learning rate mentioned in Table 1 is the rate at which weights will be updated during training. The small learning rate computational cost is less and in this study the mentioned rate was supposed to be best for the proposed model. The parameter iterations/epochs are the number of runs of ANN during training. As more epochs will be, then model will take more time and may not reduce loss further or reduce at much slower rate and chances of model overfitting. Therefore, the 4000 epochs were considered to be best for the model training and optimisation. Remaining parameters were selected as default and change in parameters may worsen the model performance.

**Table 1.** Parameter setting for the optimisation of ANN

Parameter	Setting
learning rate	0.1
beta_1	0.9
beta_2	0.999
epsilon	1 × 10^−7^
amsgrad	False
iterations/epochs	4000

## Simulations and predictions

In this section, we will discuss about proposed model evaluation and predictions to demonstrate the model performance and efficiency. Two different kinds of ANN evaluations are carried out during the whole process. First, the ANN is trained and optimised to test the efficiency of ANN and later it has been used to predict the number of reported cases in coming days in Pakistan. Numerical calculations are executed on a PC with specifications of 2.4 GHz CPU and its memory capacity is 1.5 G.

### ANN evaluation on train-test data

**Fig. 5.**
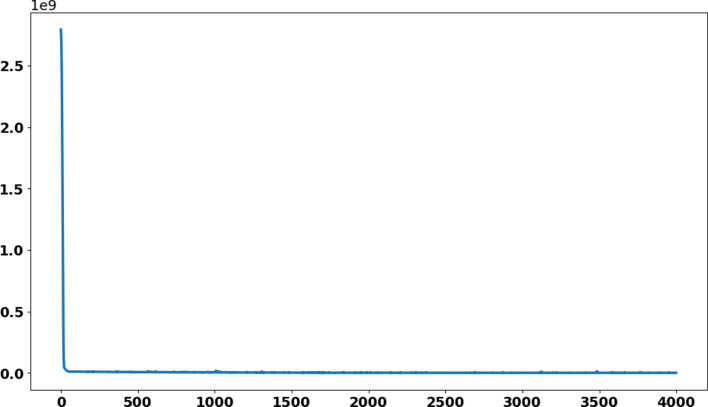
Convergence rate of loss function of ANN on train dataset.

**Fig. 6.**
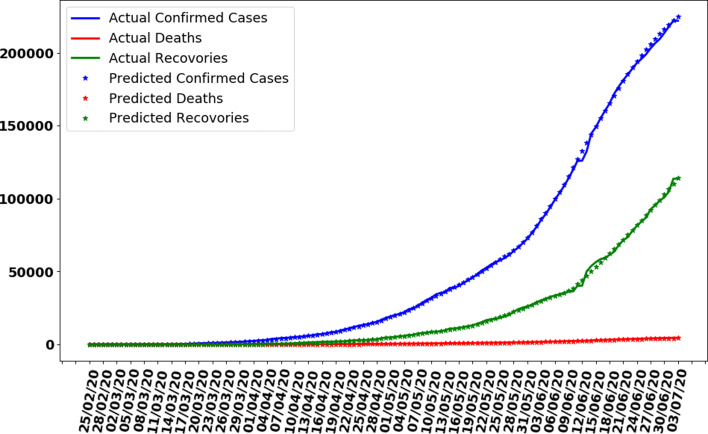
Curve fitting by ANN on training dataset.

**Fig. 7.**
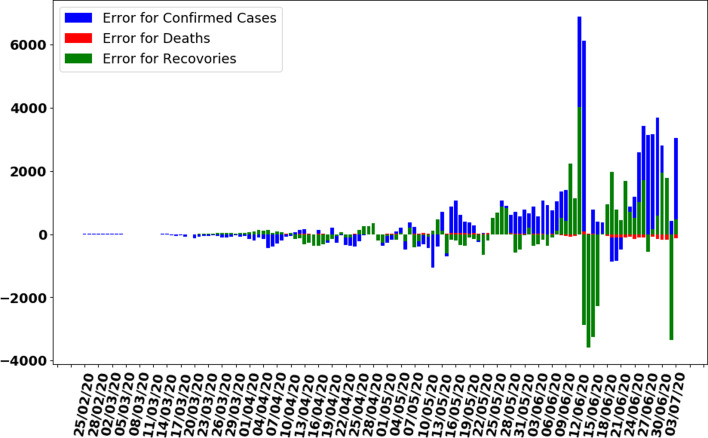
Error graph between Predicted and Actual data by ANN on training dataset.

**Fig. 8.**
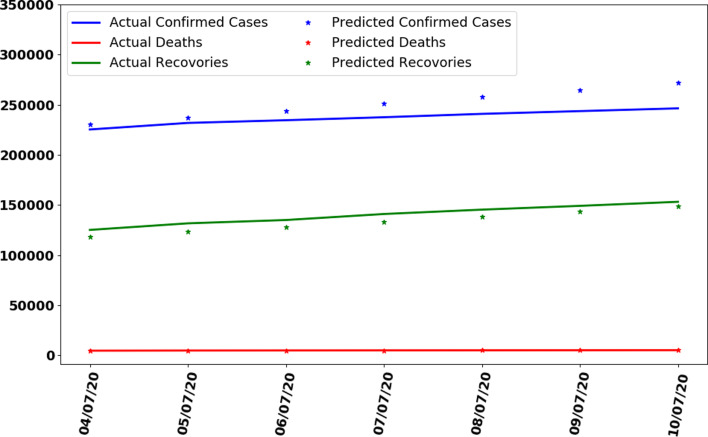
Curve fitting by trained ANN on test dataset.

**Fig. 9.**
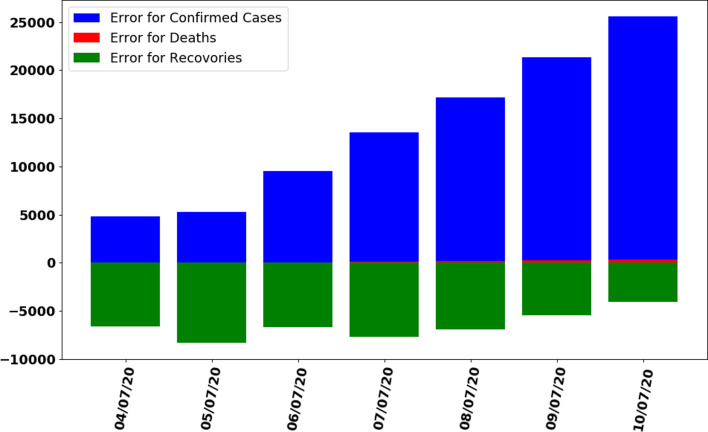
Error graph between predicted and actual data by trained ANN on test dataset.

In this section, the train-test split datasets as described in Section ‘Data gathering’ were used. The test data size was 0.05 (i.e. 5% of total data). Out of 137 total days, first 130 days data were used to train the model while next 7 days were used to test the efficiency and performance of proposed ANN to make predictions. As described in the ANN parameters and choice of the algorithm in Table 1, the convergence rate of the proposed ANN on the training dataset is shown in Figure 5 which represents the curve describing the loss function convergence rate at each iteration. It can observe that the loss function value started from 2.7 × 10^9^ and converged to 5.2 × 10^5^ in 4000 iterations (epochs). In Figure 6, the curve fitting or data fitting on actual cases is shown for all three categories i.e. confirmed cases, deaths and recoveries. It can be observed that the model is well fitted with training data and the difference between predicted results by ANN and actual results are shown in Figure 7 which represents the difference between the number of actual cases and the cases predicted by the ANN. It is observed that the error is between −1000 and 1000 up to date 09 June 2020 while there are some days on which error rise up to 4000. The calculated mean absolute error (MAE) for training data was 284. As the error graph lies between −4000 and 4000 while the output value is more than 246 351, so it can be observed that this is a good fit for making predictions of ANN. To test the efficiency and performance of the proposed ANN, trained ANN with its trained parameters was evaluated on test dataset. The graph of results predicted by the ANN and actual cases is shown in Figure 8 while the error graph representing the difference between actual and predicted cases is shown in Figure 9.

### ANN to make predictions

**Fig. 10.**
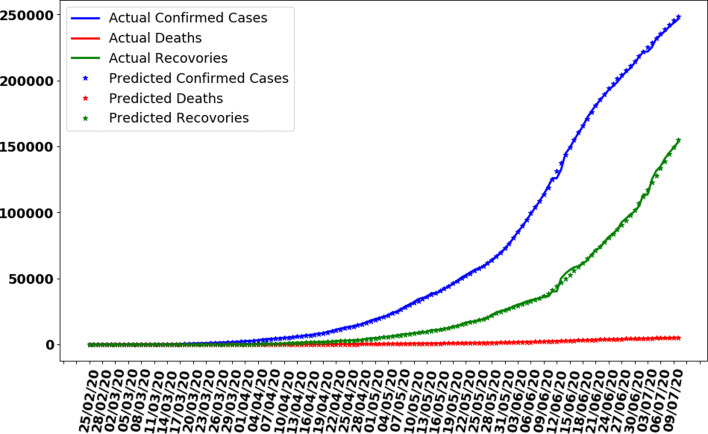
Curve fitting by trained ANN on whole data.

**Fig. 11.**
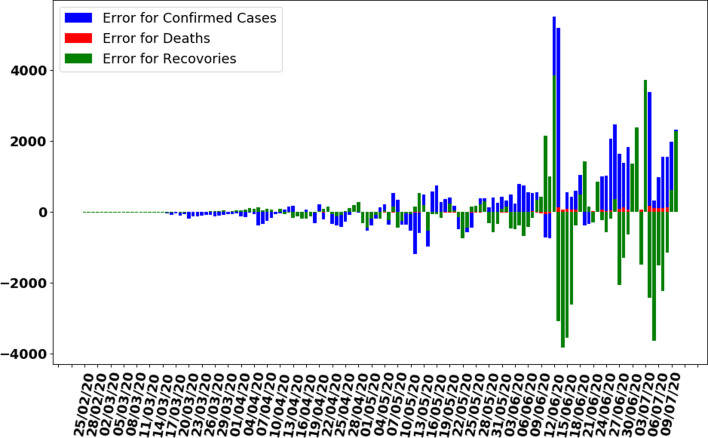
Error graph between predicted and actual data by trained ANN on whole data.

**Fig. 12.**
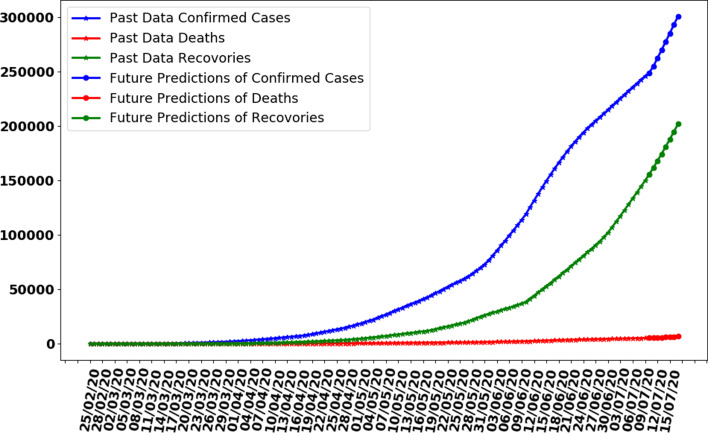
Making predictions with trained ANN for next 7 days.

In this section, whole data are used to train the proposed ANN model. The model is trained on 137 days data. The curve fitting is better than the previously trained model as the whole is used to train the model with an MAE of 335. The data are generated for the next 7 days based on previously generated input data. In Figure 10, the curve fitting on whole data is shown for all three categories. It can be observed that the model is well fitted with data and difference between predicted results by ANN and actual results is shown in Figure 11. Figure 12 represents the graph for cases of coronavirus pandemic up to 17 July 2020 i.e. including next 7 days’ predictions. The predicted case values are given in Table 2.

**Table 2.** Predictions of coronavirus cases between 11 July 2020 and 17 July 2020

Date	Confirmed cases	Deaths	Recovered
11/07/2020	252 201	4846	162 073
12/07/2020	258 964	5005	168 533
13/07/2020	265 847	5169	175 140
14/07/2020	272 733	5334	181 931
15/07/2020	279 706	5508	188 890
15/07/2020	286 743	5692	196 021
15/07/2020	293 875	5880	203 334

It can be observed that, based on previous data of coronavirus cases the trained ANN model predicting that on 17 July 2020, the number of confirmed cases will reach 293 875, deaths 5880 and recoveries 203 334. It is observed that the confirmed cases will increase by approximately 47 524 with more 50 200 recovered people while 84 661 remaining active cases.

## Conclusion and recommendations

The proposed technique for analysis of the COVID-19 coronavirus disease is presented and future predictions for the next 7 days are performed. It is observed that the model is well fitted with training data and can help to make predictions for the future. The effect of variable parameters on three different cases infected, deaths and recoveries on daily basis are shown which would help the Government for the future safety of people and to control the spread of pandemic if the same practice remains continues. In the future, a more robust ANN can design by using more input parameters collected from real life which are affecting the spread of coronavirus. Therefore, ANN would help to make decisions for controlling parameters and make more accurate policies and decisions.

## Data Availability

The whole research study which supports this study is openly available in [Predictions-of-COVID-19-in-Pakistan] at https://doi.org/10.5281/zenodo.4013997.

## Supplementary material

For supplementary material accompanying this paper visit http://dx.doi.org/10.1017/S0950268820002174. click here to view supplementary material
